# Serum LDL-C/HDL-C ratio and the risk of carotid plaques: a longitudinal study

**DOI:** 10.1186/s12872-022-02942-w

**Published:** 2022-11-24

**Authors:** Zhuchao Wu, Xiaona Li, Qin Wen, Bilin Tao, Beibei Qiu, Qun Zhang, Jianming Wang

**Affiliations:** 1 Department of Epidemiology, Center for Global Health, School of Public Health, Nanjing Medical University, 211166 Nanjing, China; 2 Department of Health Management Center, the First Affiliated Hospital of Nanjing Medical University, 210029 Nanjing, China; 3 Department of Health Management, Center for Global Health, School of Public Health, Nanjing Medical University, 211166 Nanjing, China

**Keywords:** Carotid plaque, Serum lipids, Lipid ratio, Longitudinal study

## Abstract

**Background:**

Dyslipidemia contributes to an increased risk of carotid atherosclerosis. However, the association between the ratio of low-density lipoprotein cholesterol (LDL-C) to high-density lipoprotein cholesterol (HDL-C) and carotid plaque formation has not been well documented. This study aims to assess the role of LDL-C/HDL-C in the risk of carotid plaque formation in a Chinese population.

**Methods:**

We followed 2,191 participants who attended the annual routine health examination. Cox proportional hazards regression, restricted cubic spline (RCS), and subgroup analysis were applied to evaluate the association between the LDL-C/HDL-C ratio and carotid plaques. The hazard ratio (HR) and 95% confidence interval (CI) were used to estimate the strength of the association.

**Results:**

Among 2,191 participants, 388 had incident carotid plaques detected, with a median follow-up time of 1.05 years. Compared with subjects younger than 45 years, those aged 45 to 59 years (HR: 2.00, 95% CI: 1.55–2.58) and over 60 years (HR: 3.36, 95% CI: 2.47–4.58) had an increased risk of carotid plaque formation. Males (HR: 1.26, 95% CI: 1.01–1.56), diabetes (HR: 1.46, 95% CI: 1.06–2.01) and a high LDL-C/HDL-C ratio (HR: 1.22, 95% CI: 1.07–1.38) were significantly linked with the occurrence of carotid plaques. After adjusting for potential confounding factors, we observed that a high LDL-C/HDL-C ratio promoted carotid plaque events (HR: 1.30, 95% CI: 1.12–1.50). The RCS analysis revealed a significant nonlinear association. The association was stronger among females (*P*-interaction < 0.05).

**Conclusion:**

A high LDL-C/HDL-C ratio could accelerate the occurrence of carotid plaques. Older men with diabetes and dyslipidemia are the critical target population. Women may be more likely to benefit from lipid-lowering interventions and thus avoid carotid plaque formation.

**Supplementary Information:**

The online version contains supplementary material available at 10.1186/s12872-022-02942-w.

## Introduction

Carotid atherosclerosis is a critical pathophysiological process in the progression of many cardiovascular diseases and is present in the early stage. In the long subclinical stage, the clinical manifestations and symptoms of patients depend on the presence of carotid plaques and their characteristics (stable plaques or vulnerable plaques) [1]. Carotid ultrasound can be used as a noninvasive method to study preclinical carotid atherosclerosis by detecting plaques and their characteristics [2]. Several studies have demonstrated that the formation of carotid plaques could increase the risk of ischemic strokes and other cardiovascular disease events, whose potential disease burden places enormous pressure on medical and socioeconomic systems [3–7]. Therefore, it is of great significance to identify risk factors for carotid plaque formation and provide interventions at the early stage for high-risk groups, resulting in decreased cardiovascular accidents.

Carotid plaques are formed by thickening of the arterial intima, blocking of the vascular lumen and tissue ischemia. Abnormal lipid metabolism and the inflammatory response can increase lipid deposition in the inner wall of blood vessels, consequently accelerating carotid plaque formation [8]. Dyslipidemia is generally defined as an increase in total cholesterol (TC), low-density lipoprotein cholesterol (LDL-C), and triglyceride (TG) and a decline in high-density lipoprotein cholesterol (HDL-C) [9]. The unfavorable lipid profile has been linked to the acceleration of carotid plaque formation and premature subclinical atherosclerosis [10]. The causal relationship between high levels of LDL-C and the progression of carotid atherosclerosis has been verified [11, 12]. Nonetheless, even in patients with well-controlled LDL-C levels, there are quite a few residual risks of atherosclerosis that may be attributed to triglyceride-rich lipoprotein particles [13]. High serum TG concentrations have been independently associated with subclinical carotid atherosclerosis in women [10]. It is generally believed that the possible mechanism of the protective effect of elevated HDL-C on carotid plaque formation and stroke is that HDL can transport cholesterol from the periphery for delivery back to the liver, where it is metabolized or eliminated in bile [14]. Moreover, HDL has anti-inflammatory effects and prevents oxidation, thus protecting endothelial cell functions [15, 16]. However, some researchers have questioned the causality between HDL-C and adverse cardiovascular events based on Mendelian randomization and genome-wide association studies [17, 18]. Considering the levels of LDL-C and HDL-C, the LDL-C/HDL-C ratio can better possess a greater capacity for evaluating the extent of lipid accumulation.

Recent studies have shown that the LDL-C/HDL-C ratio can be used as a new biomarker to predict the risk of several diseases and has prognostic value. Zou et al. [19] concluded, based on a large sample of longitudinal cohorts, that the LDL-C/HDL-C ratio is an independent predictor of nonalcoholic fatty liver disease. A case‒control study indicated that the LDL-C/HDL-C ratio was the main risk factor for ischemic strokes [20]. Kuang et al. [21] found that the LDL-C/HDL-C ratio also has some value in prediabetes risk assessment, and the accuracy was better than that of LDL-C and HDL-C. However, their predictive value is often applied only to subgroups of the whole population, such as nonobese people with normal lipids and patients with nonvalvular atrial fibrillation. Employing the LDL-C/HDL-C ratio as a biomarker for predicting carotid plaque occurrence is unclear. Our study aims to evaluate the relationship between the LDL-C/HDL-C ratio and carotid plaque formation in a large, longitudinal cohort of the Chinese population.

## Methods

### Study subjects

**Fig. 1:**
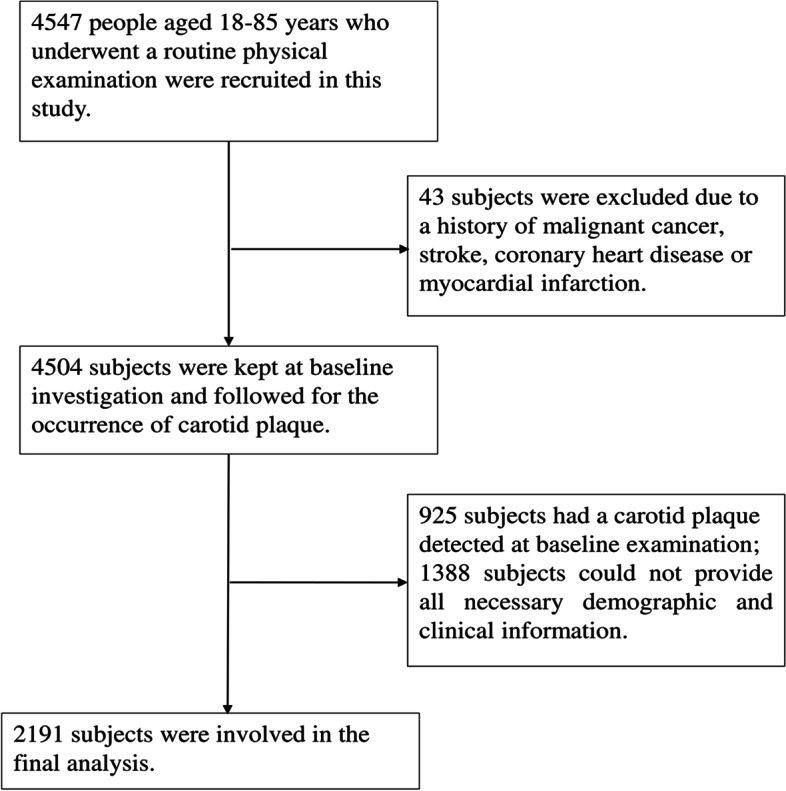
Flow chart of the selection process for eligible participants

We recruited a group of Chinese adults who underwent a regular physical examination at the Health Management Center of the First Affiliated Hospital of Nanjing Medical University from January 2017 to December 2020. The inclusion criteria of subjects were: (a) individuals aged 18–85; (b) individuals with at least two annual physical examinations; and (c) individuals whose data including serum lipids and carotid ultrasonography could be obtained. Participants were excluded if they: (a) had a previous diagnosis of cancer, stroke, coronary heart disease, or myocardial infarction; (b) had plaques detected at baseline examination; (c) took lipid-lowering drugs, or (d) could not provide necessary demographic and clinical information. Ultimately, we recruited 2191 participants (1279 males and 912 females) and their complete information is in the analyses (Fig. 1).

### Atherosclerosis assessment

A high-resolution B-mode ultrasound instrument was employed to identify carotid plaques bilaterally, which were divided into three parts of the carotid artery: the common carotid artery, the bifurcation, and the internal carotid artery [22]. The carotid plaque was described as an area whose thickness was over 1.5 mm as assessed from the media adventitia interface to the lumen-intima interface, or as a local wall whose thickness was at least 1.5 times that of the blood vessel walls surrounding it [2]. The incident carotid plaque was defined as carotid plaque newly detected during the follow-up period. Hyperechoic and isoechoic plaques were defined as stable plaques, and hypoechoic and mixed echoic plaques were defined as vulnerable plaques [22, 23]. All carotid plaque measurements followed a strict quality control procedure during image acquisition.

### Assessment of demographic, behavioral, and clinical characteristics

A standard questionnaire was designed to collect demographic characteristics, medical history, and living habits. Body mass index (BMI) was calculated by taking a participant’s weight (kg) and dividing it by his or her height squared (m^2^). Systolic blood pressure (SBP) and diastolic blood pressure (DBP) were measured by clinical staff. After an overnight fast, venous blood samples were collected to conduct biochemistry tests. TC, TG, HDL-C, and LDL-C levels were calculated by standard enzymatic methods. Meanwhile, the glycated hemoglobin A1c (HbA1c) concentration was determined by ion-exchange high-performance liquid chromatography.

### Definitions of variables

Smokers were identified as smoking one or more daily cigarettes for no less than 6 months. A former smoker was described as someone who quit smoking at least one year ago. Hypertension was identified by elevated blood pressure, either an SBP ≥ 140 mmHg or DBP ≥ 90 mmHg, and self-reported current consumption of antihypertensive drugs for hypertension [24]. Diabetes was defined following the American Diabetes Association 2020 criteria, which includes a fasting plasma glucose level of no less than 7.0 mmol/L or an HbA1c concentration of 6.5% or higher and a self-reported previous diagnosis of dieabetes [25]. Having a BMI over 25 kg/m^2^ was considered as overweight, whereas a BMI exceeding 30 kg/m^2^ was considered as obesity.

### Statistical analysis

Normally distributed continuous variables were expressed as the mean ± standard deviation, and comparisons among subgroups were analyzed by a *t test* or one-way ANOVA. Categorical variables were analyzed by a chi-squared test. Cox proportional hazard regression was performed to extensively identify the factors related to the incidence of carotid plaques. Stepwise multivariate analysis following the Akaike information criterion was employed to establish a nomogram [26]. The hazard ratio (HR) and 95% confidence interval (CI) were used to estimate the strength of the association. Meanwhile, the roles of HDL-C and LDL-C in the formation of carotid plaques were also assessed. Additionally, we compared the capacity of HDL-C, LDL-C, and the LDL-C/HDL-C ratio to forecast carotid plaque incidence by a receiver operating characteristic curve. Associations between the LDL-C/HDL-C ratio and the risk of carotid plaque formation were evaluated on a continuous scale with restricted cubic spline (RCS) curves. The count of knots was determined by assessing the AIC of the univariate models with 3, 4, and 5 knots. Probable linearity was identified by utilizing a likelihood ratio test comparing the model with only a linear item and the model with linearly added cubic spline items [27, 28]. Furthermore, stratified analyses by sex, age, hypertension, and diabetes were performed to examine the above association. The significance level α was set at 0.05, and a two-tailed test was utilized. All statistical analyses were completed in R 4.1.2 (https://www.r-project.org/).

## Results

### Baseline characteristics of participants

We followed 2191 individuals for new carotid plaque occurrence, with a median follow-up duration of 1.05 years and interquartile range (IQR) of 0.96 to 1.99 years. A total of 388 (17.71%) subjects developed carotid plaques during this period, of which 129 were vulnerable plaques. As shown in Table 1, patients with carotid plaques were more likely to be male, older, current smokers, had a higher prevalence of hypertension and diabetes, and had increased BMI, HbA1c, LDL-C, LDL-C/HDL-C ratio, TG and non-HDL-C than patients without carotid plaques (all *P* < 0.05).

**Table 1:** Baseline characteristics of subjects

Characteristics	Carotid plaque		*P*- value
	**No (** ***n*** ** = 1803)**	**Yes (** ***n*** ** = 388)**	
Gender			
Female, n(%)	778(43.15)	134(34.54)	**0.002**
Male, n(%)	1025(56.85)	254(65.46)	
Age (years), median (IQR)	44(38, 51)	51(45, 58)	**< 0.001**
< 45, n(%)	918(50.92)	83(21.39)	**< 0.001**
45–59, n(%)	732(40.60)	221(56.96)	
≥ 60, n(%)	153(8.49)	84(21.65)	
Smoking status			**0.004**
Never, n(%)	1524(84.53)	301(77.58)	
Current, n(%)	246(13.64)	78(20.10)	
Former, n(%)	33(1.83)	9(2.32)	
Hypertension, n(%)	389(21.58)	128(32.99)	**< 0.001**
Diabetes, n(%)	83(4.60)	44(11.34)	**< 0.001**
BMI (kg/m^2^), median (IQR)	23.84(21.78, 26.06)	24.35(22.65, 26.45)	**0.001**
≥ 25 kg/m^2^, n(%)	658(36.49)	158(40.72)	0.132
SBP (mmHg), median (IQR)	121.00(110.00, 132.00)	124.00(115.00, 135.00)	**< 0.001**
DBP (mmHg), median (IQR)	75.00(68.00, 84.00)	78.00(71.00, 86.00)	**< 0.001**
HbA1c(%)	5.40(5.20, 5.60)	5.50(5.30, 5.80)	**< 0.001**
TC (mmol/L), median (IQR)	5.38(4.81, 6.13)	5.51(4.79, 6.21)	0.302
LDL-C (mmol/L), median (IQR)	3.42(2.97, 3.94)	3.54(3.06, 4.03)	**0.012**
HDL-C (mmol/L), median (IQR)	1.33(1.13, 1.55)	1.26(1.10, 1.45)	**< 0.001**
LDL-C/HDL-C ratio, median (IQR)	2.61(2.05, 3.18)	2.81(2.36, 3.28)	**< 0.001**
TG (mmol/L), median (IQR)	1.33(0.94, 1.94)	1.41(1.01, 2.17)	**0.016**
Non-HDL-C (mmol/L), median (IQR)	4.04(3.47, 4.74)	4.14(3.54, 4.80)	**0.022**

Bold *P*-value indicates significance

*Abbreviation: BMI* body mass index, *SBP *systolic blood pressure, *DBP *diastolic blood pressure, *HbA1c *glycosylated hemoglobin, *TC *total cholesterol, *HDL-C *high-density lipoprotein cholesterol, *LDL-C *low-density lipoprotein cholesterol, *TG* triglyceride, IQR interquartile range

### Risk factors for carotid plaques

Univariate Cox regression analysis showed that male sex, old age, current smoking, hypertension, diabetes, BMI, SBP, DBP, HbA1c, and LDL-C/HDL-C ratio were significantly associated with carotid plaques (all *P* < 0.05). Stepwise multivariate Cox regression analysis indicated that subjects whose LDL-C/HDL-C ratio was high were more likely to develop carotid plaques (HR: 1.22, 95% CI: 1.07–1.38). Additionally, males (HR: 1.26, 95% CI: 1.01–1.56) and patients diagnosed with diabetes (HR: 1.46, 95% CI: 1.06–2.01) were more likely to develop carotid plaques. Compared with subjects aged < 45 years, those aged 45 to 59 years (HR: 2.00, 95% CI: 1.55–2.58) and ≥ 60 years (HR: 3.36, 95% CI: 2.47–4.58) had a multifold increased risk of carotid plaque formation (Table 2).

**Table 2:** Risk analysis of incident carotid plaque

Characteristics	Univariate analysis		Multivariate analysis	
	**HR (95%CI)**	***P*** **-value**	**HR(95%CI)**	***P*** **-value**
Gender				
Female	Reference		Reference	
Male	1.33(1.08, 1.64)	**0.008**	1.26(1.01, 1.56)	**0.040**
Age (years)				
< 45	Reference		Reference	
45–59	2.10(1.63, 2.71)	**< 0.001**	2.00(1.55, 2.58)	**< 0.001**
≥ 60	3.39(2.50, 4.59)	**< 0.001**	3.36(2.47, 4.58)	**< 0.001**
History of smoking				
Never	Reference			
Current	1.18(0.92, 1.51)	0.203		
Former	1.36(0.70, 2.63)	0.368		
Hypertension	1.50(1.21, 1.85)	**< 0.001**		
Diabetes	1.97(1.44, 2.70)	**< 0.001**	1.46(1.06, 2.01)	**0.021**
BMI (kg/m^2^)	1.05(1.02, 1.08)	**0.003**		
≥ 25 kg/m^2^	1.16(0.95, 1.43)	0.140		
SBP (per 20 mmHg increase)	1.25(1.11, 1.41)	**< 0.001**		
DBP (per 20 mmHg increase)	1.18(1.00, 1.40)	0.055		
HbA1c	1.38(1.21, 1.59)	**< 0.001**		
TC	1.03(0.94, 1.13)	0.551		
LDL-C/HDL-C ratio	1.29(1.15, 1.45)	**< 0.001**	1.22(1.07, 1.38)	**0.002**
TG	1.05(0.96, 1.16)	0.259		

Bold *P*-value indicates significance

*Abbreviation: BMI* body mass index, *SBP *systolic blood pressure, *DBP *diastolic blood pressure, *HbA1c *glycosylated hemoglobin, *TC *total cholesterol, *HDL-C *high-density lipoprotein cholesterol, *LDL-C *low-density lipoprotein cholesterol, *TG *triglyceride, *HR *hazard ratio, *CI *confidence interval

### LDL-C/HDL-C ratio and risk of carotid plaques

**Fig. 2:**
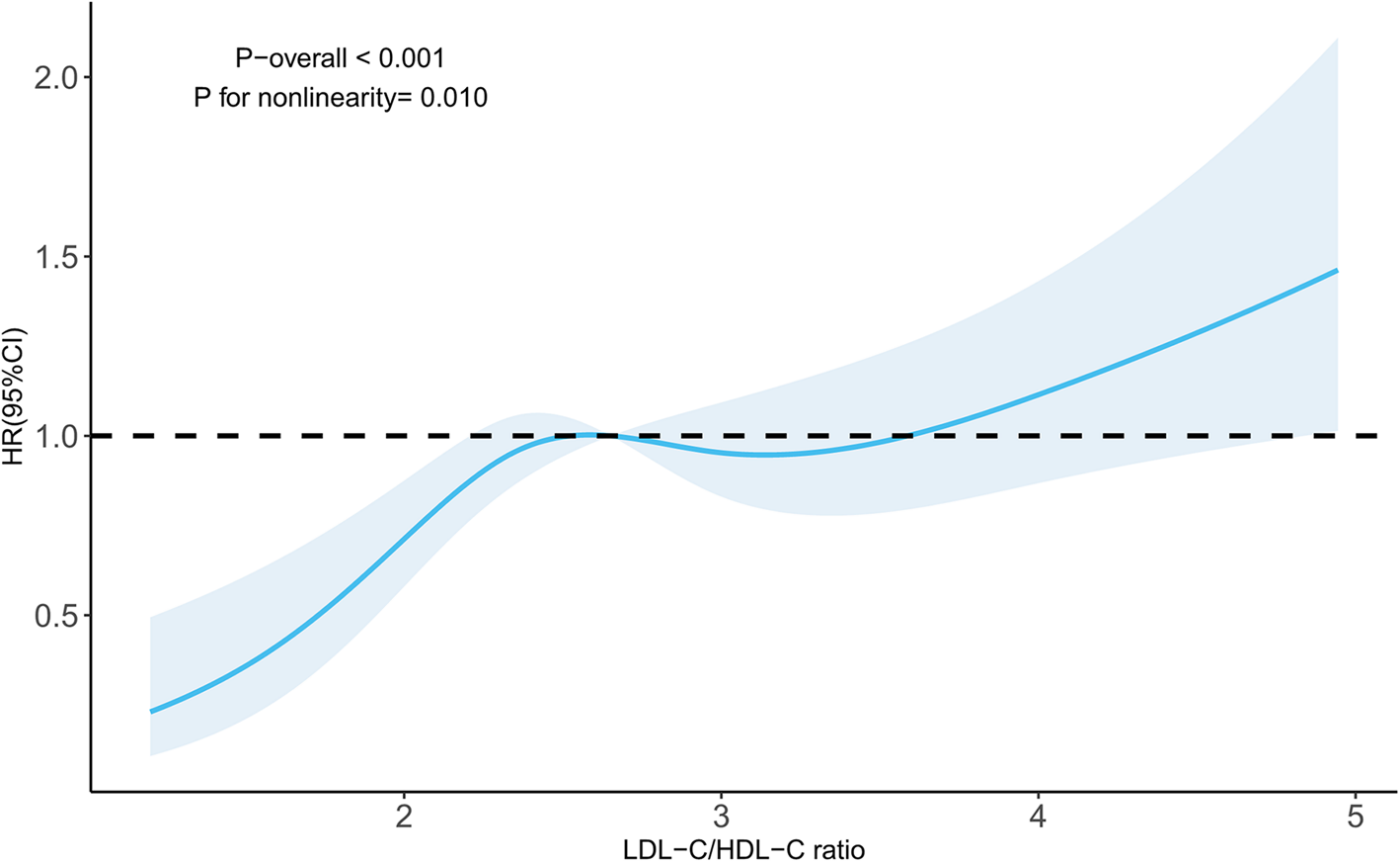
Association between the LDL-C/HDL-C ratio and the risk of new-onset carotid plaques

The relationship of the LDL-C/HDL-C ratio with the occurrence of carotid plaques is summarized in Table 3. No significant change existed in the main tendency of the LDL-C/HDL-C ratio on carotid plaque formation in all adjusted models. The LDL-C/HDL-C ratio increased by 1 mmol/L, and the risk of carotid plaque formation was augmented by 30% in the completely adjusted model. Similar analysis steps were applied to explore HDL-C and LDL-C in the subsequent analysis. The results showed that LDL-C were positively correlated with new carotid plaque formation (HR: 1.50, 95% CI: 1.01–2.24), while HDL-C was negatively correlated with carotid plaque incidence (HR: 0.45, 95% CI: 0.28–0.73). In addition, an AUC (areas under the curve) was calculated to compare the capacity of HDL-C, LDL-C, and the LDL-C/HDL-C ratio to estimate the probability of carotid plaque occurrence. The AUCs of different lipoproteins were as follows: LDL-C: 0.541 < HDL-C: 0.568 < LDL-C/HDL-C ratio: 0.580 (Supplemental Table S1). We plotted the RCS to explore the nonlinear correlation between the LDL-C/HDL-C ratio and the hazard ratio of carotid plaque occurrence, as shown in Fig. 2 (*P* for nonlinearity = 0.010 in adjusted Model 3).

**Table 3:** LDL-C/HDL-C ratio and risk of carotid plaque formation

Variables	Model 1 HR(95% CI)	Model 2 HR(95% CI)	Model 3 HR(95% CI)
LDL-C (mmol/L)	1.10(0.96, 1.26)	1.10(0.96, 1.25)	1.50(1.01, 2.24)
HDL-C (mmol/L)	0.59(0.40, 0.86)	0.62(0.42, 0.92)	0.45(0.28, 0.73)
LDL-C/HDL-C ratio	1.22(1.07, 1.38)	1.21(1.07, 1.38)	1.30(1.12, 1.50)

Model 1 was adjusted for age and sex. Model 2 was further adjusted for smoking status, hypertension, and diabetes based on Model 1. Model 3 was further adjusted for BMI, SBP, DBP, HbA1c, TC, and TG based on Model 2

*Abbreviation: BMI* body mass index, *SBP *systolic blood pressure, *DBP *diastolic blood pressure, *HbA1c *glycosylated hemoglobin, *TC *total cholesterol, *HDL-C *high-density lipoprotein cholesterol, *LDL-C *low-density lipoprotein cholesterol, *TG *triglyceride, *HR *hazard ratio, *CI *confidence interval

### Subgroup analysis and interaction test

The results of subgroup analysis by different variables, including sex, age, hypertension, and diabetes, are shown in Table 4. The analysis demonstrated that the elevated LDL-C/HDL-C ratio contributes to carotid plaque occurrence, with HRs ranging from 1.27 to 1.40. However, there was no significant correlation between carotid plaque formation and the LDL-C/HDL-C ratio among males, participants over 60 years, and patients diagnosed with diabetes and hypertension. No interaction was found except in the analyses stratified by sex (*P* for interaction = 0.021).

**Table 4:** Subgroup analysis

Variables	Model 1 HR(95% CI)	Model 2 HR(95% CI)	Model 3 HR(95% CI)	*P* for interaction
Gender				**0.021**
Female	1.43(1.25, 1.62)	1.42(1.24, 1.62)	1.40(1.21, 1.62)	
Male	1.07(0.90, 1.27)	1.12(0.94, 1.33)	1.13(0.95, 1.36)	
Age (years)				0.928
< 60	1.32(1.16, 1.49)	1.28(1.12, 1.47)	1.28(1.11, 1.47)	
≥ 60	1.26(0.95, 1.68)	1.24(0.92, 1.66)	1.26(0.93, 1.72)	
Hypertension				0.075
No	1.35(1.19, 1.53)	1.32(1.16, 1.52)	1.30(1.13, 1.50)	
Yes	1.01(0.79, 1.29)	1.04(0.81, 1.34)	1.03(0.80, 1.33)	
Diabetes				0.900
No	1.31(1.16, 1.47)	1.28(1.13, 1.46)	1.27(1.11, 1.45)	
Yes	0.86(0.59, 1.26)	0.96(0.65, 1.42)	0.98(0.66, 1.46)	

Model 1, crude model. Model 2 adjusted for sex and age. Model 3 was further adjusted for hypertension, diabetes, and smoking status based on Model 2

*P *for interaction based on Model 3

*Abbreviation:HR *hazard ratio, *CI *confidence interval

### Supplementary analysis

The AUC of lipid parameters (LDL-C, HDL-C, non-HDL-C, and LDL-C/HDL-C ratio) and the combined model (including LDL-C combined with HDL-C, LDL-C combined with LDL-C/HDL-C ratio, and HDL-C combined with LDL-C/HDL-C ratio) for predicting the presence of carotid plaques are shown in Supplemental Table S1. Baseline characteristics for patients with stable or vulnerable plaques are shown in Supplemental Table S2. The association between non-HDL-C and the risk of carotid plaque formation is shown in Supplemental Table S3. Moreover, stepwise multivariate analysis selected the LDL-C/HDL-C ratio, sex, age, and diabetes for nomogram construction (Supplemental Figure S1). Each variable was assigned a relevant point value for its role in the model. Total points can be calculated by the mathematical expression 52 (if age ≥ 60) + 9 (if male) + 16 (if diabetic) + 8* LDL-C/HDL-C ratio.

## Discussion

This study provided novel insight into the relationship between the LDL-C/HDL-C ratio and carotid plaque formation in Chinese adults. We proved that the LDL-C/HDL-C ratio was positively associated with the risk of carotid plaque formation, and RCS analysis showed a nonlinear relationship between the LDL-C/HDL-C ratio and carotid plaque formation. Subgroup analyses further identified an interaction between sex and the LDL-C/HDL-C ratio in predicting carotid plaque formation.

Vascular endothelial inflammation caused by dyslipidemia is a crucial event in the pathogenesis of atherosclerosis [29]. A prospective cohort study on a low-income Chinese population revealed that high concentrations of LDL-C were positively associated with the risk of carotid plaque formation, especially in women [30]. HDL can prevent atherosclerosis by protecting the vascular endothelium and reversing cholesterol transport function [31]. Yin et al. found that low levels of HDL-C (< 1.04 mmol/L) were significantly associated with unstable carotid plaques in populations with a high risk of stroke [32]. A case‒control study involving 187 patients with severe carotid artery stenosis revealed that the LDL-C/HDL-C ratio was an independent risk factor for vulnerable plaque [33]. However, the study of Yang et al. [34], a cross-sectional study with comparatively few subjects, did not demonstrate causality. These findings were consistent with the results of the present study. Although participants with a high LDL-C/HDL-C ratio have normal or even high levels of HDL-C, HDL may lose its antioxidant effects or acquire proinflammatory features and become dysfunctional, thereby inducing atherosclerotic progression [35]. In addition, the LDL-C/HDL-C ratio has been reported to be closely related to HDL particle distribution. An elevated LDL-C/HDL-C ratio can prohibit HDL maturation and the process of reverse cholesterol transport, consequently promoting atherosclerotic progression [36, 37]. Therefore, monitoring the LDL-C/HDL-C ratio is of great significance to identify high-risk groups for carotid plaque formation.

Older age and diabetes mellitus are known risk factors for carotid plaque formation and atherosclerosis [38, 39]. The metabolic disorders that accompany diabetes, such as chronic hyperglycemia, dyslipidemia, and insulin resistance, lead to impaired function of vascular endothelial cells, smooth muscle cells, and platelets, which predisposes patients to carotid plaques [40–42]. Researchers have reported a higher prevalence and incidence of carotid plaques in men than in women [30]. Possible reasons include the sex-specific nature of genes involved in inflammation and endothelial function [43] and sex differences in carotid bifurcation anatomy [44]. The subgroup analysis in our study also suggested that females were more likely to benefit from such lipid-regulating measures, thereby reducing the risk of carotid plaque occurrence. This is consistent with Lin et al. [30], who showed that controlling LDL-C is essential for alleviating atherosclerosis in women. This may be related to the fact that there are more risk factors for carotid plaque formation in men, and comprehensive intervention in many aspects is needed. Smoking has been proven to be closely associated with the risk of carotid plaque formation, particularly in current smokers [45, 46], but current smoking was not significant in our study, either through univariate analysis or multivariate analysis. This may be because our study did not collect pack-years of smoking (burden) or the number of cigarettes smoked per day (intensity) [46]. Therefore, clinicians should reinforce monitoring of this ratio to avoid carotid plaques, especially in elderly men with diabetes. Among the females, lowering the lipid ratio had an additional benefit in reducing the risk of carotid plaque formation.

A significant strength of this research was that it was conducted with a comparatively large sample size, prospective study design, and took into account the involvement of other potential risk factors. In addition, we conducted a comprehensive analysis of the relationship between the LDL-C/HDL-C ratio and carotid plaque formation and provided more reliable evidence for the prevention of carotid plaques in the general population.

### Limitations

Our study inevitably had certain limitations. First, the participants were recruited from one center, and the conclusions should be replicated and verified in other populations. Second, we failed to collect data on exercise habits and dietary intake, which are considered modifiable factors for carotid plaque formation [47, 48]. Finally, medications, especially lipid-lowering drugs, could also affect the development of carotid plaques [49, 50]. However, we excluded subjects with a previous diagnosis of stroke, coronary heart disease, and myocardial infarction and those with carotid plaques at baseline, which are primary indications for lipid-lowering therapy [51]. Excluding those potential drug users at baseline could mitigate the effect to some extent.

### Future directions

Risk thresholds for the LDL-C/HDL-C ratio should be established in large cohorts, identifying those at high risk for primary stroke prevention. In addition, the association between the LDL-C/HDL-C ratio and the transition from stable to vulnerable carotid plaques will further enhance clinical practice value.

## Conclusion

A high LDL-C/HDL-C ratio could accelerate the occurrence of carotid plaques. Older men with diabetes and dyslipidemia should be the critical intervention population. Women are more likely to benefit from lipid-lowering interventions and thus avoid carotid plaque formation.

## Supplementary Information

**Additional file 1: Supplemental Table S1.** AUC with the 95% CI of serum lipoproteins and LDL-C/HDL-C ratio for predicting carotid plaques. **Supplemental Table S2.** Baseline characteristics for patients with plaques. **Supplemental Table S3.** Non-HDL-C and risk of carotid plaques. **Supplemental Figure S1.** A nomogram was used to predict the risk of carotid plaques among a Chinese population.

## Data Availability

The datasets used and/or analyzed during the current study are available from the corresponding author on reasonable request.

## Abbreviations

HDL-C: High-density lipoprotein cholesterol
LDL-C: Low-density lipoprotein cholesterol
RCS: Restricted cubic spline
HR: Hazard ratio
CI: Confidence interval
TC: Total cholesterol
HbA1c: Glycated hemoglobin A1c
BMI: Body mass index
SBP: Systolic blood pressure
DBP: Diastolic blood pressure
FBG: Fasting blood glucose
TC: Total cholesterol
TG: Triglyceride
AUC: The area under the curve

## References

[1] Daghem M, Bing R, Fayad ZA, Dweck MR (2020). Noninvasive Imaging to Assess Atherosclerotic Plaque Composition and Disease Activity: Coronary and Carotid Applications. JACC Cardiovasc Imaging.

[2] Nicolaides AN, Panayiotou AG, Griffin M, Tyllis T, Bond D, Georgiou N, Kyriacou E, Avraamides C, Martin RM (2022). Arterial Ultrasound Testing to Predict Atherosclerotic Cardiovascular Events. J Am Coll Cardiol.

[3] Baradaran H, Eisenmenger LB, Hinckley PJ, de Havenon AH, Stoddard GJ, Treiman LS, Treiman GS, Parker DL, Scott McNally J. (2021). Optimal Carotid Plaque Features on Computed Tomography Angiography Associated With Ischemic Stroke. J Am Heart Assoc.

[4] Kamtchum-Tatuene J, Noubiap JJ, Wilman AH, Saqqur M, Shuaib A, Jickling GC. Prevalence of High-risk Plaques and Risk of Stroke in Patients With Asymptomatic Carotid Stenosis: A Meta-analysis. JAMA Neurol. 2020;77(12):1524–35. 10.1001/jamaneurol.2020.2658 PMC7400201 32744595

[5] Mitchell C, Korcarz CE, Gepner AD, Kaufman JD, Post W, Tracy R, Gassett AJ, Ma N, McClelland RL, Stein JH (2018). Ultrasound carotid plaque features, cardiovascular disease risk factors and events: The Multi-Ethnic Study of Atherosclerosis. Atherosclerosis.

[6] Vouillarmet J, Marsot C, Maucort-Boulch D, Riche B, Helfre M, Grange C (2019). Vascular Events and Carotid Atherosclerosis: A 5-Year Prospective Cohort Study in Patients with Type 2 Diabetes and a Contemporary Cardiovascular Prevention Treatment. J Diabetes Res.

[7] Song P, Fang Z, Wang H, Cai Y, Rahimi K, Zhu Y, Fowkes FGR, Fowkes FJI, Rudan I (2020). Global and regional prevalence, burden, and risk factors for carotid atherosclerosis: a systematic review, meta-analysis, and modelling study. Lancet Glob Health.

[8] Li Z, Cheng Q, Liu Y, Cheng X, Wang S, He Y, Wang X, Huang M, Li Y, Xue X (2021). Low-/high-density lipoprotein cholesterol ratio and carotid plaques in patients with coronary heart disease: a Chinese cohort study. Lipids Health Dis.

[9] Handelsman Y, Jellinger PS, Guerin CK, Bloomgarden ZT, Brinton EA, Budoff MJ, Davidson MH, Einhorn D, Fazio S, Fonseca VA (2020). Consensus Statement by the American Association of Clinical Endocrinologists and American College of Endocrinology on the Management of Dyslipidemia and Prevention of Cardiovascular Disease Algorithm – 2020 Executive Summary. Endocr Pract.

[10] Fanlo-Maresma M, Esteve-Luque V, Pintó X, Padró-Miquel A, Corbella E, Candás-Estébanez B (2022). Study of common hypertriglyceridaemia genetic variants and subclinical atherosclerosis in a group of women with SLE and a control group. Lupus Sci Med.

[11] Borén J, Chapman MJ, Krauss RM, Packard CJ, Bentzon JF, Binder CJ, Daemen MJ, Demer LL, Hegele RA, Nicholls SJ (2020). Low-density lipoproteins cause atherosclerotic cardiovascular disease: pathophysiological, genetic, and therapeutic insights: a consensus statement from the European Atherosclerosis Society Consensus Panel. Eur Heart J.

[12] Pan Z, Guo H, Wang Q, Tian S, Zhang X, Li C, Ma Z (2022). Relationship between subclasses low-density lipoprotein and carotid plaque. Transl Neurosci.

[13] Packard CJ (2022). Remnants, LDL, and the Quantification of Lipoprotein-Associated Risk in Atherosclerotic Cardiovascular Disease. Curr Atheroscler Rep.

[14] Koch M, Aroner SA, Fitzpatrick AL, Longstreth WT, Furtado JD, Mukamal KJ, Jensen MK (2022). HDL (High-Density Lipoprotein) Subspecies, Prevalent Covert Brain Infarcts, and Incident Overt Ischemic Stroke: Cardiovascular Health Study. Stroke.

[15] Rader DJ, Alexander ET, Weibel GL, Billheimer J, Rothblat GH (2009). The role of reverse cholesterol transport in animals and humans and relationship to atherosclerosis. J Lipid Res.

[16] Rysz J, Gluba-Brzózka A, Rysz-Górzyńska M, Franczyk B (2020). The Role and Function of HDL in Patients with Chronic Kidney Disease and the Risk of Cardiovascular Disease. Int J Mol Sci.

[17] Lind L, Ingelsson M, Sundstrom J, Ärnlöv J (2021). Impact of risk factors for major cardiovascular diseases: a comparison of life-time observational and Mendelian randomisation findings. Open Heart.

[18] Casula M, Colpani O, Xie S, Catapano AL, Baragetti A (2021). HDL in Atherosclerotic Cardiovascular Disease: In Search of a Role. Cells.

[19] Zou Y, Zhong L, Hu C, Zhong M, Peng N, Sheng G (2021). LDL/HDL cholesterol ratio is associated with new-onset NAFLD in Chinese non-obese people with normal lipids: a 5-year longitudinal cohort study. Lipids Health Dis.

[20] Zhang XX, Wei M, Shang LX, Lu YM, Zhang L, Li YD, Zhang JH, Xing Q, Tu-Erhong ZK, Tang BP (2020). LDL-C/HDL-C is associated with ischaemic stroke in patients with non-valvular atrial fibrillation: a case-control study. Lipids Health Dis.

[21] Kuang M, Peng N, Qiu J, Zhong Y, Zou Y, Sheng G (2022). Association of LDL:HDL ratio with prediabetes risk: a longitudinal observational study based on Chinese adults. Lipids Health Dis.

[22] Iannuzzi A, Rubba P, Gentile M, Mallardo V, Calcaterra I, Bresciani A, Covetti G, Cuomo G, Merone P, Di Lorenzo A (2021). Carotid Atherosclerosis, Ultrasound and Lipoproteins. Biomedicines.

[23] Khan F, Gonçalves I, Shore AC, Natali A, Palombo C, Colhoun HM, Östling G, Casanova F, Kennbäck C, Aizawa K (2022). Plaque characteristics and biomarkers predicting regression and progression of carotid atherosclerosis. Cell Rep Med.

[24] Unger T, Borghi C, Charchar F, Khan NA, Poulter NR, Prabhakaran D, Ramirez A, Schlaich M, Stergiou GS, Tomaszewski M (2020). 2020 International Society of Hypertension Global Hypertension Practice Guidelines. Hypertension.

[25] American Diabetes A (2020). 2. Classification and Diagnosis of Diabetes: Standards of Medical Care in Diabetes-2020. Diabetes Care.

[26] Balachandran VP, Gonen M, Smith JJ, DeMatteo RP (2015). Nomograms in oncology: more than meets the eye. Lancet Oncol.

[27] Durrleman S, Simon R (1989). Flexible regression models with cubic splines. Stat Med.

[28] Govindarajulu US, Spiegelman D, Thurston SW, Ganguli B, Eisen EA (2007). Comparing smoothing techniques in Cox models for exposure-response relationships. Stat Med.

[29] Tang M, Zhao Q, Yi K, Wu Y, Xiang Y, Cui S, Su X, Yu Y, Zhao G, Jiang Y (2022). Association between four nontraditional lipids and ischemic stroke: a cohort study in Shanghai, China. Lipids Health Dis.

[30] Lin Y, Li Y, Li Z, Zhang Z, Liu J, Sun J, Tu J, Wang J, Zhang W, Li J (2022). Sex-Related Differences in the Incidence and Development of Carotid Plaques in a Low-Income Chinese Population-A Prospective Cohort Study. Int J Womens Health.

[31] Hu X, Liu J, Li W, Wang C, Li G, Zhou Y, Dong H (2022). Elevated serum uric acid was associated with pre-inflammatory state and impacted the role of HDL-C on carotid atherosclerosis. Nutr Metab Cardiovasc Dis.

[32] Yin J, Yu C, Liu H, Du M, Sun F, Yu C, Wei L, Wang C, Wang X (2020). A model to predict unstable carotid plaques in population with high risk of stroke. BMC Cardiovasc Disord.

[33] Liu H, Chen Z, Ding J, MaiMaiTi S, Cai J, Qiao T. Relationship of the low-density lipoprotein cholesterol/high-density lipoprotein cholesterol ratio with vulnerable plaque in patients with severe carotid artery stenosis: A case-control study in the Han Chinese population. Curr Neurovasc Res. 2022;19(2):160–70. 10.2174/1567202619666220629160733 35770391

[34] Yang C, Sun Z, Li Y, Ai J, Sun Q, Tian Y (2014). The correlation between serum lipid profile with carotid intima-media thickness and plaque. BMC Cardiovasc Disord.

[35] Bonacina F, Pirillo A, Catapano AL, Norata GD (2021). HDL in Immune-Inflammatory Responses: Implications beyond Cardiovascular Diseases. Cells.

[36] Tian L, Liu Y, Qin Y, Long S, Xu Y, Fu M (2010). Association of the low-density lipoprotein cholesterol/high-density lipoprotein cholesterol ratio and concentrations of plasma lipids with high-density lipoprotein subclass distribution in the Chinese population. Lipids Health Dis.

[37] Saleheen D, Scott R, Javad S, Zhao W, Rodrigues A, Picataggi A, Lukmanova D, Mucksavage ML, Luben R, Billheimer J (2015). Association of HDL cholesterol efflux capacity with incident coronary heart disease events: a prospective case-control study. Lancet Diabetes Endocrinol.

[38] Zhan C, Wang Q, Chen Z, Pang H, Tu J, Ning X, Wang J, Fei S, Ji X (2022). Association of metabolic syndrome with carotid atherosclerosis in low-income Chinese individuals: A population-based study. Front Cardiovasc Med.

[39] Woolsey AB, Arsang-Jang S, Spence JD, Hackam DG, Azarpazhooh MR (2022). The impact of socioeconomic status on the burden of atherosclerosis, and the effect of intensive preventive therapy on its progression: A retrospective cohort study. Atherosclerosis.

[40] Lupu L, Taha L, Farkash R, Bayya F, Karmi M, Steinmetz Y, Shaheen FF, Perel N, Hamayel K, Levi N (2022). Hemoglobin A1C as a prognostic factor and the pre-diabetic paradox in patients admitted to a tertiary care medical center intensive cardiac care unit: The Jerusalem platelets thrombosis and intervention in cardiology (JUPITER-6) study group. Cardiovasc Diabetol.

[41] Liu R, Li L, Shao C, Cai H, Wang Z (2022). The Impact of Diabetes on Vascular Disease: Progress from the Perspective of Epidemics and Treatments. J Diabetes Res.

[42] Hu X, Li W, Wang C, Zhang H, Lu H, Li G, Zhou Y, Dong H (2021). Association between the Plasma-Glycosylated Hemoglobin A1c/High-Density Lipoprotein Cholesterol Ratio and Carotid Atherosclerosis: A Retrospective Study. J Diabetes Res.

[43] Li J, Zhang P, Yi X, Luo H, Yu M, Chen H, Wang C (2022). Sex-specific association between inflammation and endothelial function relevant gene and vulnerable carotid plaque. Front Physiol.

[44] Schulz UG, Rothwell PM (2001). Sex differences in carotid bifurcation anatomy and the distribution of atherosclerotic plaque. Stroke.

[45] Ji X, Leng XY, Dong Y, Ma YH, Xu W, Cao XP, Hou XH, Dong Q, Tan L, Yu JT (2019). Modifiable risk factors for carotid atherosclerosis: a meta-analysis and systematic review. Ann Transl Med.

[46] Kianoush S, Yakoob MY, Al-Rifai M, DeFilippis AP, Bittencourt MS, Duncan BB, Bensenor IM, Bhatnagar A, Lotufo PA, Blaha MJ (2017). Associations of Cigarette Smoking With Subclinical Inflammation and Atherosclerosis: ELSA-Brasil (The Brazilian Longitudinal Study of Adult Health). J Am Heart Assoc.

[47] Fiuza-Luces C, Santos-Lozano A, Joyner M, Carrera-Bastos P, Picazo O, Zugaza JL, Izquierdo M, Ruilope LM, Lucia A (2018). Exercise benefits in cardiovascular disease: beyond attenuation of traditional risk factors. Nat Rev Cardiol.

[48] Coates AM, Hill AM, Tan SY (2018). Nuts and Cardiovascular Disease Prevention. Curr Atheroscler Rep.

[49] Hussain MA, Saposnik G, Raju S, Salata K, Mamdani M, Tu JV, Bhatt DL, Verma S, Al-Omran M (2018). Association Between Statin Use and Cardiovascular Events After Carotid Artery Revascularization. J Am Heart Assoc.

[50] Katakami N, Mita T, Irie Y, Takahara M, Matsuoka TA, Gosho M, Watada H, Shimomura I (2018). Effect of sitagliptin on tissue characteristics of the carotid wall in patients with type 2 diabetes: a post hoc sub-analysis of the sitagliptin preventive study of intima-media thickness evaluation (SPIKE). Cardiovasc Diabetol.

[51] Mach F, Baigent C, Catapano AL, Koskinas KC, Casula M, Badimon L, Chapman MJ, De Backer GG, Delgado V, Ference BA (2020). 2019 ESC/EAS Guidelines for the management of dyslipidaemias: lipid modification to reduce cardiovascular risk. Eur Heart J.

