# Supplementary material for: Serum LDL-C/HDL-C ratio and the risk of carotid plaques: a longitudinal study
Source: BMC Cardiovasc Disord. 2022 Nov 24;22:501. doi: 10.1186/s12872-022-02942-w (PMC9700971; doi:10.1186/s12872-022-02942-w)
Supplement: Supplementary file 1 — Additional file 1: Supplemental Table S1. AUC with the 95% CI of serum lipoproteins and LDL-C/HDL-C ratio for predicting carotid plaques. Supplemental Table S2. Baseline characteristics for patients with plaques. Supplemental Table S3. Non-HDL-C and risk of carotid plaques. Supplemental Figure S1. A nomogram was used to predict the risk of carotid plaques among a Chinese population. [file 12872_2022_2942_MOESM1_ESM.docx]

**Supplemental Table S1.** AUC with the 95%CI of serum lipoproteins and LDL-C/HDL-C ratio for predicting carotid plaques

| **Variables** | **AUC (95% CI)** | **Specificity** | **Sensitivity** |
| --- | --- | --- | --- |
| LDL-C (mmol/L) | 0.541(0.510,0.572) | 0.537 | 0.541 |
| HDL-C (mmol/L) | 0.568(0.537,0.598) | 0.418 | 0.709 |
| LDL-C/HDL-C ratio | 0.580(0.550,0.609) | 0.359 | 0.791 |
| LDL-C combined with HDL-C | 0.581(0.551, 0.610) | 0.345 | 0.799 |
| LDL-C combined with LDL-C/HDL-C ratio | 0.581(0.552, 0.610) | 0.325 | 0.825 |
| HDL-C combined with LDL-C/HDL-C ratio | 0.580(0.550,0.609) | 0.382 | 0.765 |
| Non-HDL-C (mmol/L) | 0.537(0.506,0.568) | 0.188 | 0.881 |

**Abbreviations:**AUC, the area under the curve; CI, confidence interval; HDL-C, high-density lipoprotein cholesterol; LDL-C, low-density lipoprotein cholesterol.

**Supplemental Table S2.** Baseline characteristics for patients with plaques

| **Characteristics** | **Carotid plaque** | | ***P*-value** |
| --- | --- | --- | --- |
|  | **Stable (n=259)** | **Vulnerable (n=129)** |  |
| Gender |  |  |  |
| Female, n(%) | 88(33.98) | 46(35.66) | 0.830 |
| Male, n(%) | 171(66.02) | 83(64.34) |  |
| Age (years), median (IQR) | 50(45, 58) | 51(46, 57) | 0.960 |
| <45, n(%) | 57(22.01) | 26(20.16) | 0.452 |
| 45-59, n(%) | 142(54.83) | 79(61.24) |  |
| ≥60, n(%) | 60(23.17) | 24(18.60) |  |
| Smoking status |  |  | 0.356 |
| Never, n(%) | 200(77.22) | 101(78.29) |  |
| Current, n(%) | 51(19.69) | 27(20.93) |  |
| Former, n(%) | 8(3.09) | 1(0.78) |  |
| Hypertension, n(%) | 84(32.43) | 44(34.11) | 0.829 |
| Diabetes, n(%) | 32(12.36) | 12(9.30) | 0.469 |
| BMI (kg/m^2^), median (IQR) | 24.44(22.62, 26.46) | 24.16(22.76, 26.33) | 0.939 |
| ≥25 kg/m^2^, n(%) | 107(41.31) | 51(39.53) | 0.821 |
| SBP (mmHg), median (IQR) | 125.00 (136.00, 137.00) | 124.00(115.00, 132.00) | 0.120 |
| DBP (mmHg), median (IQR) | 79.00(71.00, 86.00) | 77.00(70.00, 84.00) | 0.174 |
| HbA1c(%) | 5.50(5.30, 5.80) | 5.50(5.30, 5.70) | 0.429 |
| TC (mmol/L), median (IQR) | 5.47(4.83, 6.15) | 5.60(4.75, 6.40) | 0.495 |
| LDL-C (mmol/L), median (IQR) | 3.57(3.08, 3.96) | 3.49(3.02, 4.19) | 0.687 |
| HDL-C (mmol/L), median (IQR) | 1.25(1.08, 1.48) | 1.27(1.11, 1.41) | 0.694 |
| LDL-C/HDL-C ratio, median (IQR) | 2.89(2.35, 3.29) | 2.73(2.40, 3.26) | 0.970 |
| TG (mmol/L), median (IQR) | 1.39(0.97, 2.15) | 1.45(1.14, 2.20) | 0.102 |

**Abbreviations:** BMI, body mass index; SBP, systolic blood pressure; DBP, diastolic blood pressure; HbA1c, glycosylated hemoglobin; TC, total cholesterol; HDL-C, high-density lipoprotein cholesterol; TG, triglyceride; HR, hazard ratio; CI, confidence interval.

**Supplemental Table S3.** Non-HDL-C and risk of carotid plaques

| **Variable** | **Model 1**  **HR (95% CI)** | **Model 2**  **HR (95% CI)** | **Model 3**  **HR (95% CI)** |
| --- | --- | --- | --- |
| Non-HDL-C (mmol/L) | 1.07(0.97, 1.19) | 1.07(0.96, 1.18) | 1.88(1.16, 3.05) |

**Note:** Model 1 was adjusted for age and sex. Model 2 was further adjusted for smoking status, hypertension, and diabetes based on Model 1. Model 3 was further adjusted for BMI, SBP, DBP, HbA1c, TC, and TG based on Model 2.

**Abbreviations:** BMI, body mass index; SBP, systolic blood pressure; DBP, diastolic blood pressure; HbA1c, glycosylated hemoglobin; TC, total cholesterol; HDL-C, high-density lipoprotein cholesterol; TG, triglyceride; HR, hazard ratio; CI, confidence interval.


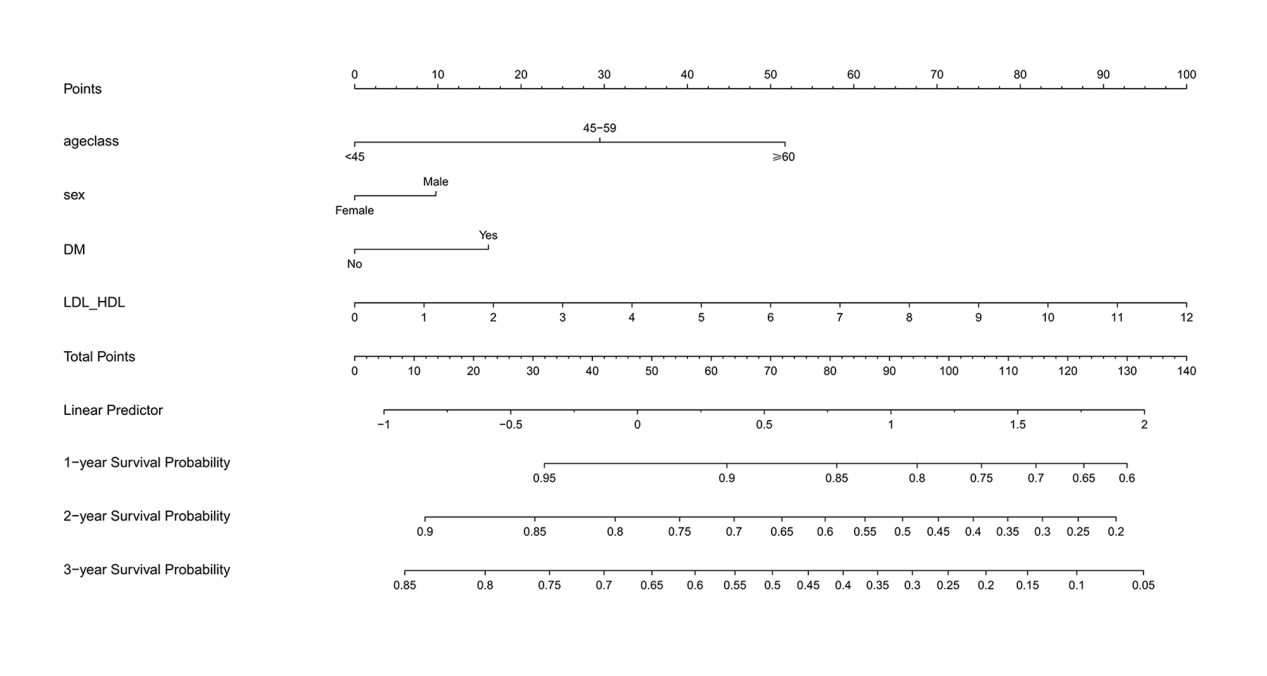


**Supplemental Figure S1.** A nomogram was used to predict the risk of carotid plaques among a Chinese population.

**Abbreviations:** HDL-C, high-density lipoprotein cholesterol; LDL-C, low-density lipoprotein cholesterol; DM, diabetes mellitus.
